# Myths of the Low-Carbohydrate Diet “Myths”. Comment on Teicholz et al. Myths and Facts Regarding Low-Carbohydrate Diets. *Nutrients* 2025, *17*, 1047

**DOI:** 10.3390/nu18213596

**Published:** 2026-10-30

**Authors:** Rami Salim Najjar

**Affiliations:** Division of Cardiology, School of Medicine, Emory University, Atlanta, GA 30307, USA; rnajjar@emory.edu

The opinion paper published by Nina Teicholz et al. [1] ignores the scientific consensus on key biological variables related to the effects of low-carbohydrate diets in cardiovascular disease (CVD) and type 2 diabetes. Additionally, the authors are methodologically inconsistent, stating that observational studies cannot reliably establish cause and effect, while nevertheless citing epidemiological evidence when those associations favor low-carbohydrate diets. Several claims characterized by the authors as “myths” are addressed below.

(I) Authors claim that the consistent rise in low-density lipoprotein cholesterol (LDL-C) observed in those consuming low-carbohydrate diets due to saturated fat intake is of little concern, and that LDL-C is not convincingly linked to CVD. This conclusion is not aligned with the current scientific consensus. The American Heart Association presidential panel has concluded that “Taking into consideration the totality of evidence, LDL cholesterol links saturated fat and its replacement macronutrients to CVD by very strong scientific evidence that satisfies rigorous criteria for causality. Three independent guidelines committees rated this evidence as Level A, Strong.” [2] In fact, no other risk factor is required for the development of atherosclerosis except elevated LDL-C [3].
(II) Authors conflate refined and unrefined carbohydrates. Replacing equal calories from saturated fat with whole grains reduces the risk of CVD, while replacing it with refined grains does not improve CVD risk [2]. It is unequivocal that whole grains, whole fruits, and beans (carbohydrate-rich foods) reduce CVD risk [4,5,6]. In fact, no evidence in the literature shows that these foods increase CVD risk.
(III) Authors fundamentally misunderstand insulin resistance by claiming that a low-carbohydrate diet can reverse type 2 diabetes. This stems from the falsehood that lowering blood glucose by removing dietary carbohydrates constitutes reversal. In reality, this reflects carbohydrate abstinence, not reversal of the underlying insulin-resistant state. Insulin resistance is driven by lipotoxicity [7], namely intramyocellular lipid accumulation promoted by diets that elevate circulating free fatty acids, such as low-carbohydrate diets. In fact, a ketogenic diet can induce insulin resistance in previously insulin-sensitive humans, whereas a high-carbohydrate diet, in contrast, favorably affects postprandial glucose [8,9]. High-carbohydrate diets derived from unrefined foods improve insulin sensitivity in diabetes based on the existing literature, and, in weight-maintaining conditions, have produced marked glycemic improvement with major reductions in insulin requirements [10], demonstrating that improvement in the underlying insulin-resistant state is achieved with unrefined high-carbohydrate diets, not by carbohydrate restriction.

Taken together, the evidence presented by Teicholz et al. [1] does not substantiate low-carbohydrate diets as a safe or disease-preventing long-term strategy for cardiometabolic health. On the contrary, evidence indicates that such diets promote mechanisms central to cardiovascular disease and type 2 diabetes. In contrast, dietary patterns rich in unrefined carbohydrates and minimally processed plant foods remain the consensus dietary approach for reducing cardiometabolic risk.

## Data Availability

No new data were created or analyzed in this study. Data sharing is not applicable to this article.
